# Supplementary material for: COCOA.jl—a Julia package for high-performance analysis of concordance and kinetic modules in biochemical networks
Source: Bioinformatics. 2026 Sep 28;42(10):btag724. doi: 10.1093/bioinformatics/btag724 (PMC13656755; doi:10.1093/bioinformatics/btag724)
Supplement: Supplementary file 1 [file btag724_supplementary_data.pdf]

Supplementary Material

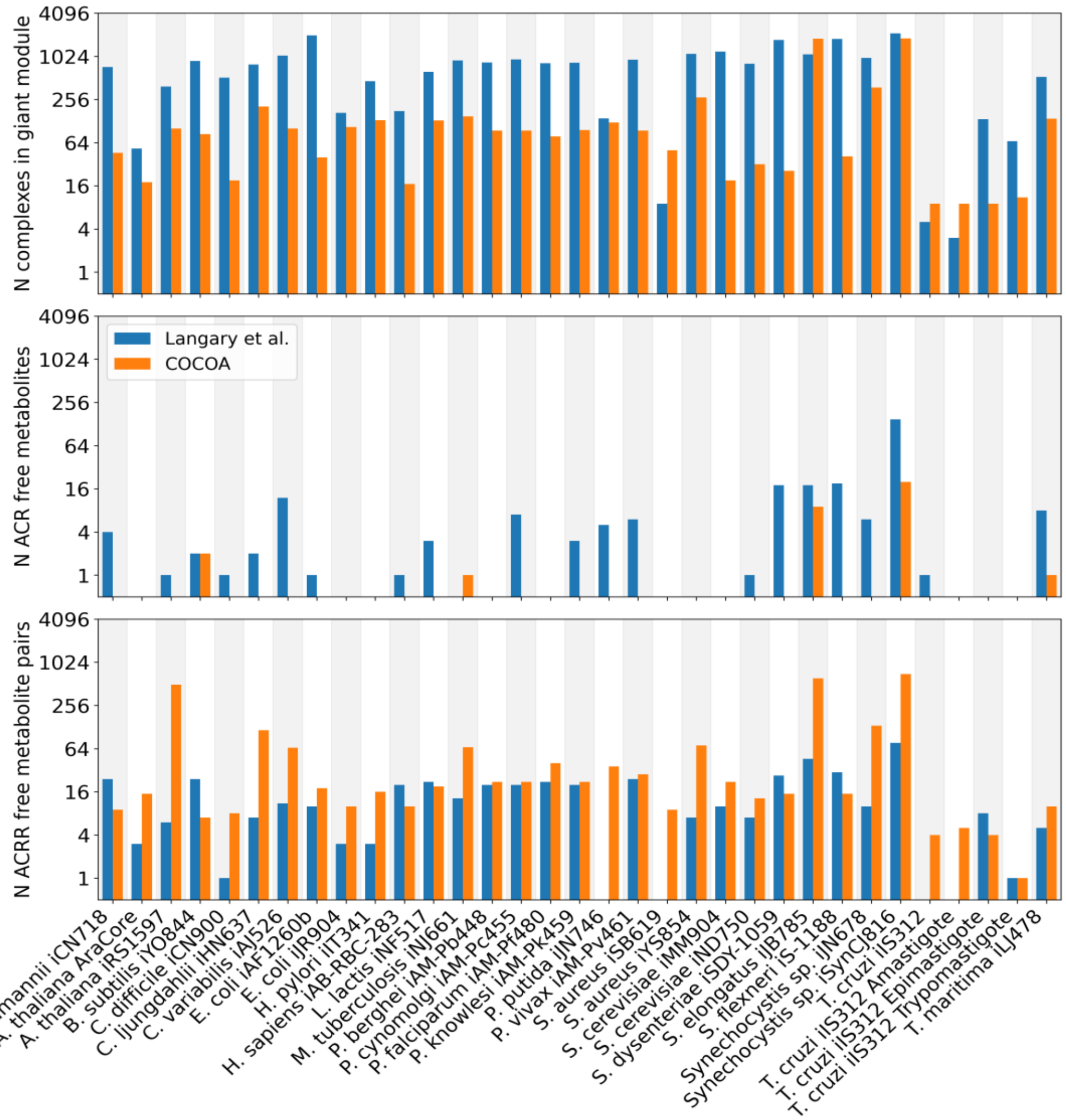

Supplementary Fig. 1. Comparison of properties based on kinetic modules for ordered binding models. Shown are the giant kinetic module size (top), the number of ACR free metabolites (middle) and the number of ACRR free metabolite pairs (bottom) for the 33 genome-scale models of Langary et al. (2025) under ordered-binding elementary-step expansion. Blue: reference MATLAB implementation (counts match Table S1 of the cited paper exactly); orange: COCOA.jl. Y-axis is log4-scaled to accommodate the three orders of magnitude spanned by the giant-module sizes.

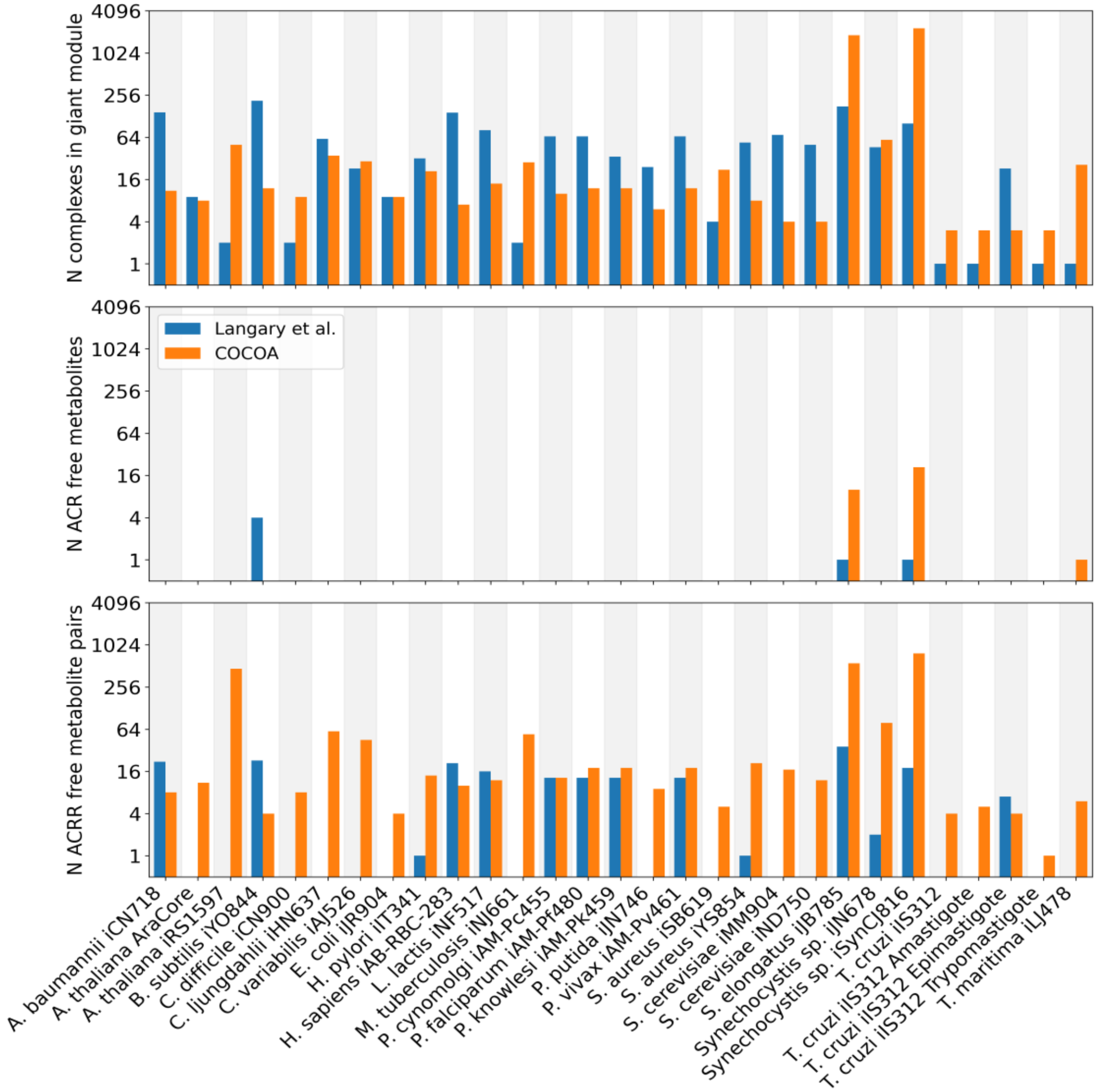

Supplementary Fig. 2. Comparison of properties based on kinetic modules for random binding models. The giant kinetic module size (top), the number of ACR free metabolites (middle) and the number of ACRR free metabolite pairs (bottom) for 29 genome-scale models of Langary et al. (2025) under random-binding elementary-step decomposition. Blue: reference MATLAB implementation; orange: COCOA.jl. Y-axis is log4-scaled to accommodate the three orders of magnitude spanned by the giant-module sizes.

Supplementary Table 1. Reproducibility and sensitivity of COCOA.jl. Seeds vary within each row. The ACR count is invariant at every setting, and the giant kinetic module is stable up to a single identifiable complex: it is 4 on e_coli_core in every run, and 106 on iJR904 except in the two seeds where one boundary complex (dkmpp_c together with three waters), whose sampled activity ratio lies at the coefficient-of-variation pre-filter threshold, is drawn in to give 107. On iAF1260b it is 40 for every seed with transitivity off and in all but one seed with it on, where the transitivity filter merges a 41-complex concordance module with the 37 balanced complexes to give 78. The concordance partition itself varies from run to run — a single partition on e_coli_core over the full cone, and distinct partitions at genome scale (on e_coli_core it also varies on the constrained biomass face and at a concordance tolerance of 10^{-10}, below solver precision) — while the ACRR count reflects finite-sampling density and converges as the flux cone is sampled more densely (iJR904: 1418 → 1378 pairs from 1000 to ≥ 5000 samples). ACR and ACRR denote the numbers of ACR metabolites and ACRR metabolite pairs; CV, coefficient of variation.

(a) e_coli_core (defaults: full steady-state cone, concordance tolerance 10^{-2}, blocked-reaction tolerance 10^{-9}, 1000 samples).

Flux space	Conc. tol.	Blocked-rxn tol.	Transitivity	Runs	Concordance modules	Distinct partitions	Giant kinetic	ACR	ACRR
full cone	10^{-2}	10^{-9}	on, off	20	109	1	4	0	1
biomass face (0.999)	10^{-2}	10^{-9}	on	3	122	3	4	0	1
full cone	10^{-6}	10^{-9}	on	3	109	1	4	0	1
full cone	10^{-8}	10^{-9}	on	3	109	1	4	0	1
full cone	10^{-10}	10^{-9}	on	3	107–109	3	4	0	1
full cone	10^{-2}	10^{-6}	on	3	109	1	4	0	1
full cone	10^{-2}	10^{-8}	on	3	109	1	4	0	1
full cone	10^{-2}	10^{-10}	on	3	109	1	4	0	1

(b) iJR904 (genome scale; full steady-state cone, concordance tolerance 10^{-2}).

Sample size	CV threshold	Transitivity	Runs	Concordance modules	Distinct partitions	Giant kinetic	ACR	ACRR
1000	0.01	on, off	17	664–676	13	106–107	0	1418–1460
5000	0.01	on	1	684	1	106	0	1378
20000	0.01	on	3	682–683	3	106	0	1378
1000	0.05	on	1	679	1	126	0	1896

(c) iAF1260b (genome scale; full steady-state cone, concordance tolerance 10^{-2}, seed × transitivity; one further run was discarded because its preprocessed network differed).

Transitivity	Runs	Concordance modules	Distinct partitions	Giant kinetic	ACR	ACRR
on	10	1994–2002	10	40–78	0	770–1327
off	8	1998–2001	8	40	0	770–846
